# Leucine, autoimmune diseases, and circulating mediators shape the causal landscape of lymphoid malignancies

**DOI:** 10.3389/fimmu.2026.1934057

**Published:** 2026-09-11

**Authors:** Dongguo Liang, Yan Ren, Yuqi Feng, Qiong Li, Amit Sharma, Ingo G.H. Schmidt-Wolf, Jingjing Pu

**Affiliations:** 1 Shanghai Institute of Hematology, National Research Center for Translational Medicine, State Key Laboratory of Medical Genomics, Ruijin Hospital, Shanghai Jiao Tong University School of Medicine, Shanghai, China; 2 Department of Hematology, Renji Hospital, Shanghai Jiao Tong University School of Medicine, Shanghai, China; 3 Shanghai Immune Therapy Institute, Renji Hospital, Shanghai Jiao Tong University School of Medicine, Shanghai, China; 4 State Key Laboratory of Digital Medical Engineering, School of Biological Science and Medical Engineering, Southeast University, Nanjing, Jiangsu, China; 5 Department of Integrated Oncology, Center for Integrated Oncology (CIO) Bonn, University Hospital Bonn, Bonn, NRW, Germany

**Keywords:** leucine, lymphoid malignancies (LM), Mendelian randomization (MR), metabolic reprogramming, risk factor, UK Biobank

## Abstract

**Introduction:**

While genetic abnormalities underlying lymphoid malignancies (LM) are well-characterized, the environmental and metabolic determinants driving LM remain largely unexplored. Autoimmune diseases are known risks, yet the specific circulating mediators and potential protective dietary factors linking these conditions to LM require systematic elucidation.

**Objectives:**

This study aimed to comprehensively screen for causal risk factors of LM, identify the metabolic mediators bridging autoimmune diseases and LM, and validate the therapeutic potential of leucine as a protective factor.

**Methods:**

We conducted a systematic Mendelian randomization analysis using 10,090 GWAS traits obtained from the IEU OpenGWAS project, including 2,568 plasma proteins and 662 plasma metabolites, to identify potential causal determinants of LM. Findings were validated using the large-scale UK Biobank cohort (n=502,175) and further supported by multi-omics data (plasma proteomics (Olink; n = 53,014) and metabolomics (NMR; 486,813)) within the same population. Mechanisms were further verified via *in vitro* assays on LM cell lines and *in vivo* experiments using a MH/NRASG12D B-cell precursor acute lymphoblastic leukemia (BCP-ALL) mouse model.

**Results:**

We identified autoimmune diseases-specifically hyperthyroidism and membranous nephropathy, as novel risk factors for LM. Mediation analysis revealed that promotilin, monounsaturated fatty acids (MUFAs), and glucose serves as key circulating mediators driving this risk. Conversely, leucine emerged as a protective metabolic factor. High-leucine conditions suppressed proliferation and induced apoptosis in LM cell lines, while dietary leucine supplementation prolonged survival and alleviated anemia and thrombocytopenia in the BCP-ALL mouse models. In patient cohorts, higher plasma leucine levels were associated with improved outcomes in non-Ph B-ALL and prolonged survival in CAR-T-treated B-cell lymphoma patients. Mechanistically, leucine exposure induced metabolic reprogramming characterized by the suppression of lipid biosynthesis pathways and reduced *SREBF1/FASN*-mediated *de novo* lipogenesis; pharmacological *FASN* inhibition recapitulated the anti-tumor effects of leucine.

**Conclusion:**

Autoimmune diseases increase LM susceptibility through specific metabolic mediators. Leucine functions as a potent protective agent and prognostic biomarker. These findings suggest that dietary leucine supplementation represents a promising and readily translatable therapeutic strategy for managing LM and enhancing CAR-T therapy efficacy.

## Introduction

Lymphoid hematopoiesis is essential for maintaining proper bodily functions, and disruptions in its differentiation or abnormal proliferation can lead to lymphoid malignancies (LM), including lymphoid leukemia and lymphoma. Originating from the lymphoid lineage, these malignancies share common genetic features, such as *KMT2A* rearrangements and *TCF3::HLF* fusions (1). Based on the cell of origin and differentiation stage, LM can be further categorized into various subtypes, such as chronic lymphocytic leukemia (CLL), acute lymphocytic leukemia (ALL), Hodgkin lymphoma (HL), and non-Hodgkin lymphoma (NHL). LM present with largely obscure etiology in many settings and rank among the top ten cancer types in global cancer morbidity and mortality statistics, exhibiting a rising trend in recent years and imposing a substantial burden on the socio-economy (2, 3). Currently, while research has extensively mapped the genomic landscape and advanced therapeutic interventions, a critical gap remains in comprehensively understanding the multifaceted environmental and metabolic drivers of tumorigenesis.

LM is caused by a combination of genetic and environmental factors. Evidence has accumulated to suggest the pivotal role of germline mutations as risk factors for LM (4, 5). Conditions such as Down syndrome (6), ataxia-telangiectasia (7), and Li-Fraumeni syndrome (8) have been associated with increased susceptibility to LM. Beyond genetic abnormalities, cohort studies have long implicated environmental exposures in the development of LM. High levels of radiation and exposure to certain chemicals, including the solvent benzene and chemotherapeutic agents, are also associated with an augmented risk of these malignancies (9, 10). Notably, some types of autoimmune diseases, comprising more than 80 diseases, have been linked to hematopoietic malignancy via observational studies, such as systemic lupus erythematosus (SLE) and lymphoma (11). However, traditional epidemiological approaches are often limited by confounding factors, reverse causation, and prohibitive costs, leaving the comprehensive causal landscape of LM largely undefined.

Mendelian randomization (MR) has emerged as a transformative strategy to dissect causal etiologies by utilizing genetic variants as naturally randomized instruments, thereby circumventing the confounding and reverse causation that plague traditional observational studies (12–14). In the specific context of LM, however, the application of MR remains fragmented and narrow. While recent studies have successfully employed MR to validate specific hypotheses, such as the links between inflammatory bowel disease (IBD) (15), celiac disease (16), sicca syndrome (17), and circulating inflammatory cytokines (18), and lymphoma, these investigations have been largely restricted to isolated subtypes and pre-defined risk factors. Consequently, a systematic, hypothesis-free screening that encompasses the full spectrum of LM, particularly lymphoid leukemia, is notably absent. Adopting such a comprehensive framework is essential to impartially identify novel drivers and metabolic mediators that traditional epidemiology may overlook.

Metabolic reprogramming, particularly the upregulation of fatty acid synthesis and glucose metabolism, is a critical hallmark of lymphoid malignancies that supports malignant proliferation (19). This metabolic dependency suggests that systemic alterations in circulating metabolites, potentially driven by underlying autoimmune conditions, could serve as key mediators of tumorigenesis. In stark contrast to these pro-tumorigenic fuels, leucine, an essential branched-chain amino acid, has emerged as a potential metabolic regulator capable of inducing autophagy and enhancing immune surveillance (20). Despite its known ability to arrest growth in other cancers, the specific potential of Leucine to act as a protective counter-measure against the metabolically dysregulated landscape of autoimmune-associated LM has not been systematically characterized.

In this study, we integrated a systematic MR analysis of 10,090 GWAS traits with large-scale epidemiological validation (UK Biobank) to screen for causal risk factors and mediators of LM. We identified novel autoimmune risks and pinpointed metabolic mediators, including promotilin and glucose. Furthermore, we combined multi-omics analysis with *in vivo* and *in vitro* functional experiments to reveal the therapeutic potential of leucine. Our findings provide a comprehensive understanding of the causal landscape of LM and highlight dietary leucine supplementation as a promising intervention strategy.

## Methods

### Mendelian randomization and meta-analysis

#### Study design and data sources

This study integrated large-scale Mendelian randomization, population-based validation, meta-analysis, and functional experiments to investigate the causal determinants and mediators of lymphoid malignancies. Summary-level GWAS data for exposures and outcomes were obtained from European populations. A total of 10,090 traits were analyzed, including 2,568 plasma proteins and 662 metabolites. Sixteen autoimmune disease entities were evaluated in the Mendelian randomization analyses. GWAS summary statistics for lymphoid malignancies were retrieved from IEU OpenGWAS and FinnGen (21).

#### Mendelian randomization analysis

Two-sample univariable Mendelian randomization (UVMR) was conducted using genome-wide significant SNPs (P < 5×10^-8^, LD r² < 0.001, 10,000 kb window). Inverse-variance weighting (IVW) was applied as the primary method, with MR-Egger and weighted median analyses used for sensitivity testing (22–24). Horizontal pleiotropy was assessed using the MR-Egger intercept. Results were considered robust when effect directions were consistent across methods and no pleiotropy was detected (P > 0.05).

#### Meta-analysis of autoimmune diseases and lymphoma risk

A meta-analysis following PRISMA guidelines was conducted using observational studies published between 2010 and 2023 (25). PubMed was searched (2010–2023) for observational studies on ulcerative colitis (UC) (26–32) or SLE (11, 27, 33–49) in relation to lymphoma. Included studies reported multivariate-adjusted OR, HR, RR, or SIR with 95% CIs; exclusions applied to case reports, non-human studies, editorials, reviews, and duplicates. Data extracted included author, year, effect sizes, and CIs. Study quality was assessed independently via the Newcastle-Ottawa Scale (NOS). Conflicts were resolved by discussion. Combined ORs with 95% CIs were estimated using DerSimonian & Laird random-effects (50). Heterogeneity was assessed by Q-test (P < 0.10) and I² statistic (51). Analyses used the R package *meta* (52) (v6.5.0) in R (v4.1.1). Statistical significance was set at P < 0.05.

### Multi-omics and clinical validation in human cohorts

#### Epidemiological and omics analysis in UK Biobank

We utilized the UK Biobank (UKB), a large-scale biomedical cohort of ~500,000 participants, for validation. First, the incidence of lymphoid leukemia, HL, DLBCL, and total lymphoma was assessed across various non-malignant diseases (defined by ICD-10 codes), with statistical significance evaluated using the chi-square test. To further cross-validate our MR findings at the molecular level, we analyzed UKB plasma proteomics (n = 53,014) and metabolomics (n ≈ 280,000) data. Differential levels of proteins and metabolites between cases and matched healthy controls were determined using two-tailed t-tests, followed by metabolite pathway enrichment analysis using the MNet R package (v0.1.0).

#### Survival analysis of LM patients

Clinical validation was performed using RNA-seq data retrieved from published cohorts (ALL and DLBCL) (53–57) and the TARGET database (https://www.cancer.gov/ccg/research/genome-sequencing/target) (58). Patients were stratified into high- and low-expression groups based on the median gene expression levels. Kaplan-Meier survival curves were generated using the survival (59, 60) (v3.5.5) and survminer (v0.4.9) (61).

### *In vitro* functional validation

Cell Culture and Leucine Treatment Human B-cell precursor leukemia (NALM-6) and diffuse large B-cell lymphoma (SU-DHL-8) cell lines were used. NALM-6 and SU-DHL-8 cells were cultured in standard RPMI 1640 medium containing 50 mg/L leucine as the basal condition (Control) or in RPMI 1640 supplemented with additional L-leucine to a final concentration of 550 mg/L (Treatment) (TargetMol, T4772), corresponding to a 10-fold increase relative to the basal medium.

#### Cell apoptosis and proliferation assays

To evaluate the cellular response to long-term leucine exposure, we performed longitudinal functional assessments. Apoptosis rates were quantified via flow cytometry using the PE Annexin V Apoptosis Detection Kit during routine medium refreshments (every 3–5 days). Following six weeks of continuous culture, cell proliferation kinetics were evaluated by seeding cells (5×10³/well) into 96-well plates, with dynamic growth monitoring performed every six hours using the Incucyte^®^ S3 Live Cell Analysis System (Sartorius).

#### Transcriptome sequencing and data analysis

Total RNA was extracted from NALM-6 cells using Vazyme RNA isolator (R401-01). Paired-end sequencing was performed on the BGI-2000 platform. Transcripts were quantified using Salmon and analyzed for differential expression with DESeq2 (62). Pathway enrichment (KEGG and GSEA) was visualized using an open-source platform (63).

### *In vivo* animal experiments

C57BL/6 mice were randomly assigned to normal-diet (Control, n = 9) and high-leucine-diet (n = 10) groups. The high-leucine diet (Ready Bite^®^, AIN-93G supplemented with 66 g/kg L-leucine) contained reduced corn starch to maintain equal caloric content. After one week, 1×10^6^ spleen cells from *MH/NRAS*^G12D^ BCP-ALL mice were injected via tail vein. Peripheral blood was collected for routine analysis (20 µL PB supplemented with 120 µl Cellpack^®^ DCL), following standard protocols. Mice were monitored regularly, and humane endpoints were predefined according to institutional guidelines. Animals reaching predefined humane endpoints were humanely euthanized. Survival distributions were estimated using Kaplan–Meier analysis and compared between groups using the log-rank test.

### Data availability

The epidemiological cohorts, plasma proteomics and plasma metabolomics are available from the UK Biobank upon application (https://www.ukbiobank.ac.uk). The data for RNAseq of NALM6 cell lines is available in the Genome Sequence Archive for Human, GSA-Human (subHRA011833).

### Ethical compliance

The study adhered to the principles outlined in the Declaration of Helsinki, and informed consent was obtained from all participants. All animal experiments in this study were conducted using mice and were approved by the Animal Ethics Committee of Ruijin Hospital (Approval No. RJ2024061). All procedures were carried out in accordance with the guidelines for the care and use of laboratory animals issued by the Ministry of Science and Technology of the People’s Republic of China. Efforts were made to minimize animal suffering and reduce the number of animals used.

## Results

### Systematic profiling of risk factors for lymphoid malignancies

**Figure 1:**
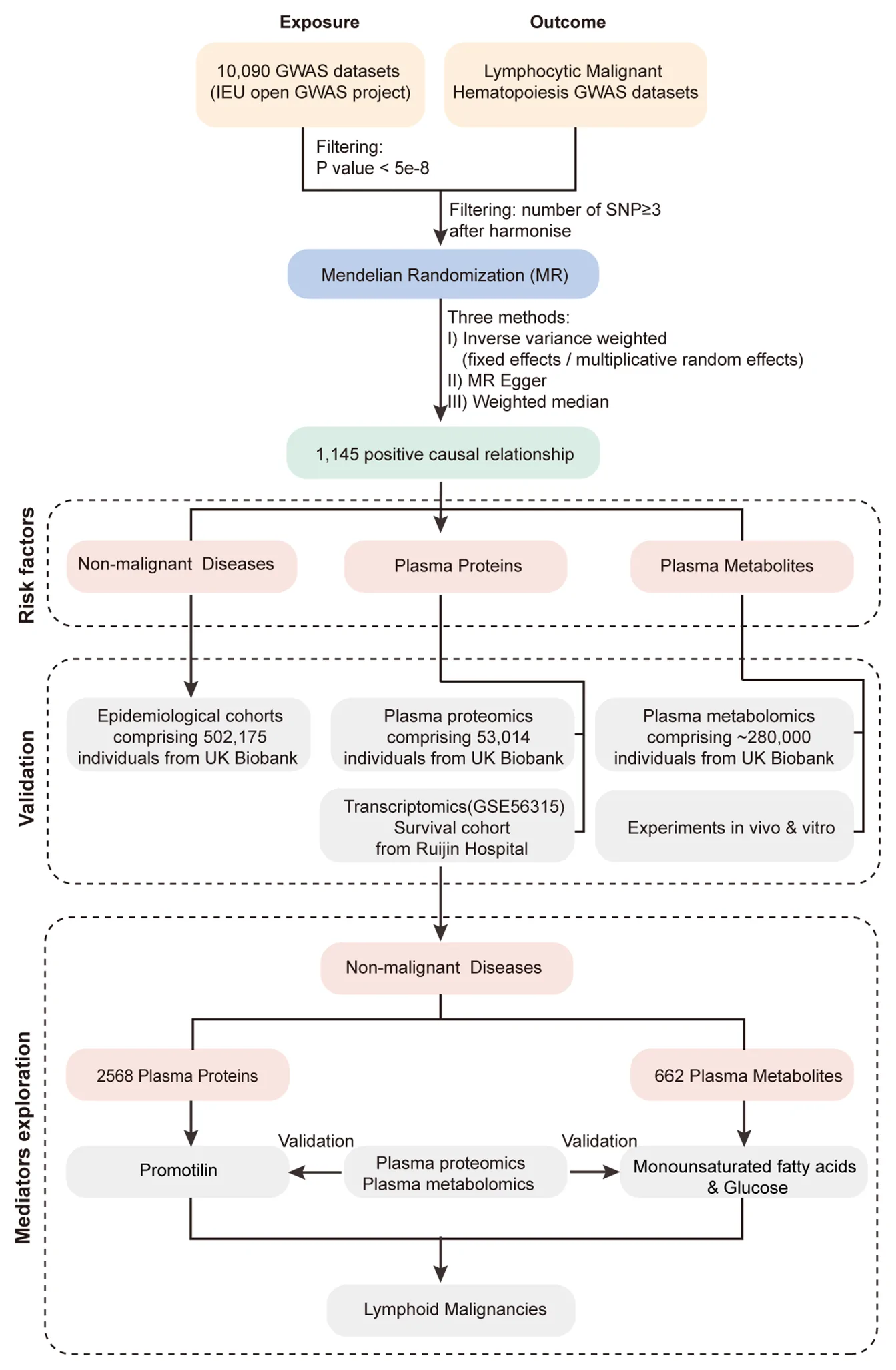
Analyzing typical exposure for lymphoid malignancies. **(A)** Experimental Workflow Diagram. This diagram outlines our investigation into the causal relationship between the exposure factor (10,090) and lymphoid hematopoiesis. Our findings were validated through large epidemiological cohorts, multi-omics data and both *in vivo* and *in vitro* experiments.

To systematically investigate the risk factors influencing the occurrence of lymphoid malignant hematopoiesis, we utilized accessible GWAS traits to conduct causal analysis using two-sample Mendelian randomization. Five accessible GWAS cohorts, including two cohorts of lymphoid leukemia (21, 64)and three cohorts of lymphoma (21, 65), were used as outcomes. In contrast, 10,090 GWAS traits obtained from the Integrative Epidemiology Unit (IEU) open GWAS project, which compiles data from the UK Biobank, FinnGen, eQTLGen, GWAS catalog-EMBL-EBI, Biobank Japan, and other databases, were used as exposure factors (**Figure 1A**). The lymphoma cohorts collected in this study include a cohort for lymphoma as a whole, a HL cohort, and a DLBCL cohort (**Table 1**), while no subtype-specific GWAS traits for lymphocytic leukemia were available to date.

**Table 1:** GWAS cohorts for outcomes in Mendelian randomization analysis.

Outcome (ICD10)	Sample size	SNPs	Consortium	Ancestry	Author	Year
Lymphoid leukemia (C91.0, C91.1, C91.3, C91.4, C91.5, C91.9)	372,776 (760/372,016)	9,015,063	UK Biobank	European	Burrows	2021
Lymphoid leukemia (C91.0, C91.1, C91.3, C91.4, C91.5, C91.6, C91.7, C91.8, C91.9)	301,445 (1,493/299,952)	20,168,082	FinnGen	European	Mitja	2023
Lymphoma (C81, C82, C83, C84, C85, C86)	361,194 (1,752/359,442)	10,226,672	UK Biobank	European	Neale lab	2018
Hodgkin lymphoma (C81)	300,732(780/299,952)	20,168,053	FinnGen	European	Mitja	2023
Diffuse non-Hodgkin lymphoma (C83)	302,554 (2,602/299,952)	20,168,109	FinnGen	European	Mitja	2023

The 10,090 GWAS traits can be divided into four major categories, including non-malignant diseases, plasma proteins, and plasma metabolites, and other traits. Following restricting to European-ancestry GWAS datasets and applying SNP-level harmonization and instrument availability criteria, 2,728 exposures were retained for MR analysis. In total, 1,145 positive exposures associated with LM were identified (**Supplementary Table 1**). We next analyzed the characteristics of these exposures and their interrelationships.

### Autoimmune diseases, particularly hyperthyroidism and membranous nephropathy, are distinct causal drivers of lymphoid malignancies

**Figure 2:**
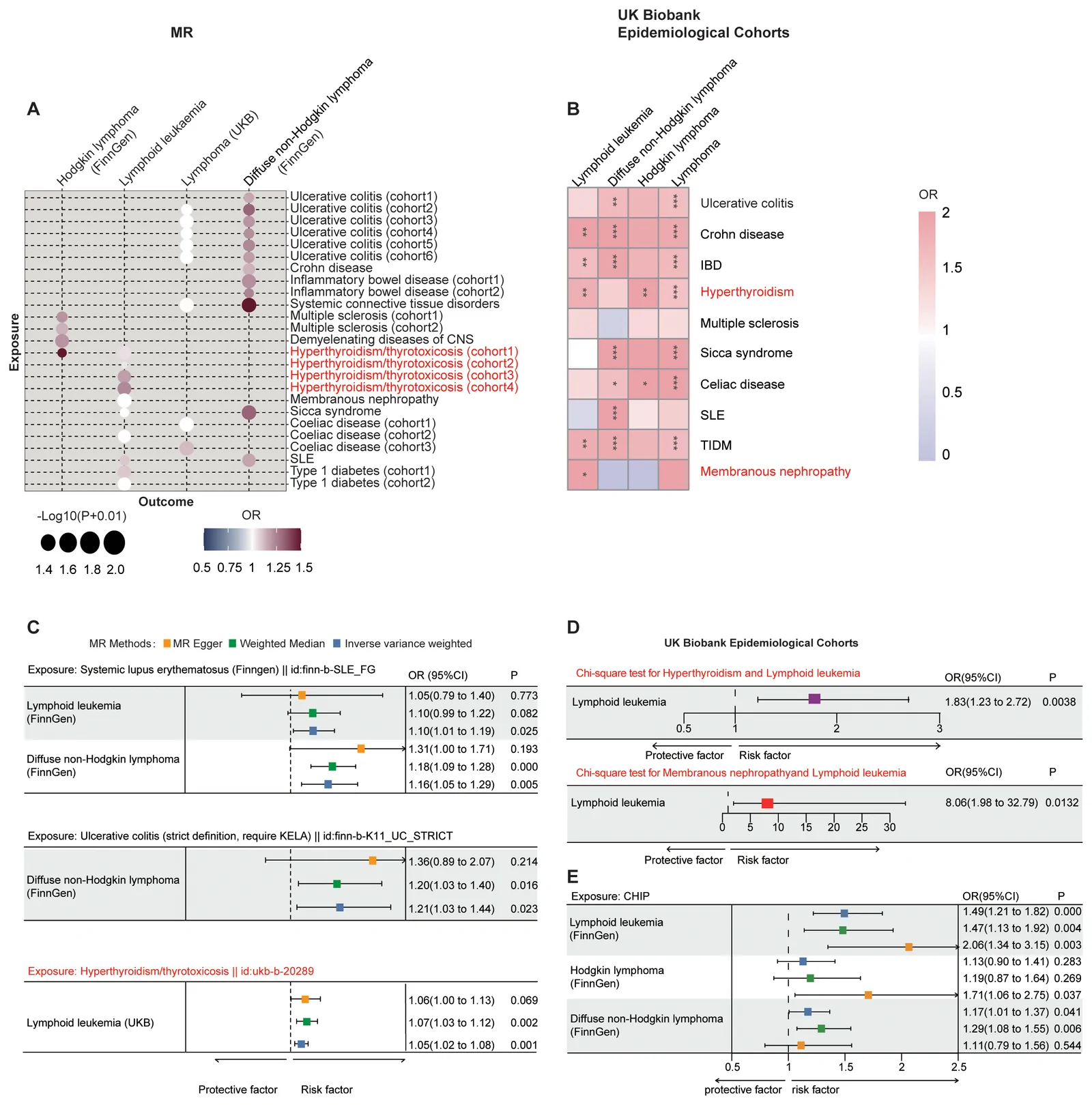
Autoimmune diseases are linked to an elevated risk of lymphoid malignancies. **(A)** Depicts the causal relationship identified by MR between autoimmune diseases and lymphoid malignancies. The complete table is listed in **Supplementary Table 1**. The circular size represents -Log10(P + 0.01) values, with colors indicating OR (Odds Ratio) values. Red signifies OR > 1, with darker shades indicating larger OR values, while blue indicates OR < 1, with darker shades indicating smaller OR values. OR larger than 1.5 is set as 1.5, while OR smaller than 0.5 will be set as 0.5. **(B)** Analysis of large observational cohorts from the UK Biobank indicates that various non-malignant diseases, especially autoimmune disorders, are positively associated with an increased incidence of lymphoid malignancies. Red signifies OR > 1, with darker shades indicating larger OR values, while blue indicates OR < 1, with darker shades indicating smaller OR values. OR larger than 2 is set as 2. * equals *p* < 0.05, ** means *p* < 0.01, while *** indicates *p* < 0.001. **(C)** Utilizes a forest plot to delineate key risk factors associated with lymphoid malignancies. The selected presentation showcases risk factors pertinent to malignant hematogenesis, including SLE, ulcerative colitis and hyperthyroidism. We employed three analytical methods: MR Egger (orange), Weighted Median (green), and Inverse Variance Weighted (blue), displaying OR values and 95% confidence intervals. **(D)** The forest plots show the positive correlations between hyperthyroidism and lymphoid leukemia, as well as membranous nephropathy and lymphoid leukemia. **(E)** Causal relationship between CHIP and LM. Three analysis methods, MR Egger (orange), Weighted Median (green), and Inverse variance weighted **(blue)** were employed. The results are presented with OR values and 95% confidence intervals. IBD: inflammatory bowel disease; SCTDs: systemic connective tissue disorders; Demyelinatingdiseases of CNS: Demyelinatingdiseases of the central nervous system.

Through Mendelian randomization, we identified numerous previously reported risk exposures related to LM. Notably, we observed significant causal associations between various autoimmune diseases and LM, including ulcerative colitis (UC), type 1 diabetes mellitus (T1DM), SLE, Sicca syndrome, multiple sclerosis (MS), coeliac disease, systemic connective tissue disorders, and demyelinating diseases of the central nervous system (CNS), confirming the reliability of our study (**Figures 2A,C**). Our meta-analysis also confirmed significant associations between autoimmune diseases and LM, showing that UC and SLE significantly increase lymphoma risk (**Supplementary Figures 1A,B**). Furthermore, we found previously unreported causal associations between certain autoimmune diseases and LM, specifically hyperthyroidism and membranous nephropathy.

We then validated these findings by analyzing large epidemiological cohorts comprising 502,175 individuals from the UK Biobank. Consistent with the Mendelian randomization results, most autoimmune diseases were associated with an increased risk of LM (**Figure 2B**). Moreover, despite some associations not showing significant differences in the Mendelian randomization analysis, our analysis using UK Biobank data revealed significant correlations between these autoimmune diseases and both lymphoma and lymphoid leukemia, complementing the Mendelian randomization findings. The newly identified risks of hyperthyroidism and membranous nephropathy for LM were also validated, with hyperthyroidism significantly increasing the risk of lymphoid leukemia and lymphoma with odds ratio of 1.83 (95% CI: 1.23-2.72, *P* = 0.0038) and 1.62 (95% CI: 1.26-2.08, *P* = 0.00017). Similarly, membranous nephropathy significantly elevated the risk of lymphoid leukemia with an OR of 8.06 (95% CI: 1.98-32.79, *P* = 0.0132) (**Figures 2B,D**).

As an exploratory analysis, in addition to autoimmune diseases, we additionally evaluated clonal hematopoiesis, an age-related biological process that may represent a parallel pathway relevant to hematopoietic disease development. Although clonal hematopoiesis is primarily associated with myeloid malignancies (66), we also found that clonal hematopoiesis was significantly associated with an increased risk of lymphoid malignancies, including lymphocytic leukemia and lymphoma (**Figure 2E**).

### Genetically predicted plasma proteomic profiling identifies specific oncogenic and protective proteins in LM

**Figure 3:**
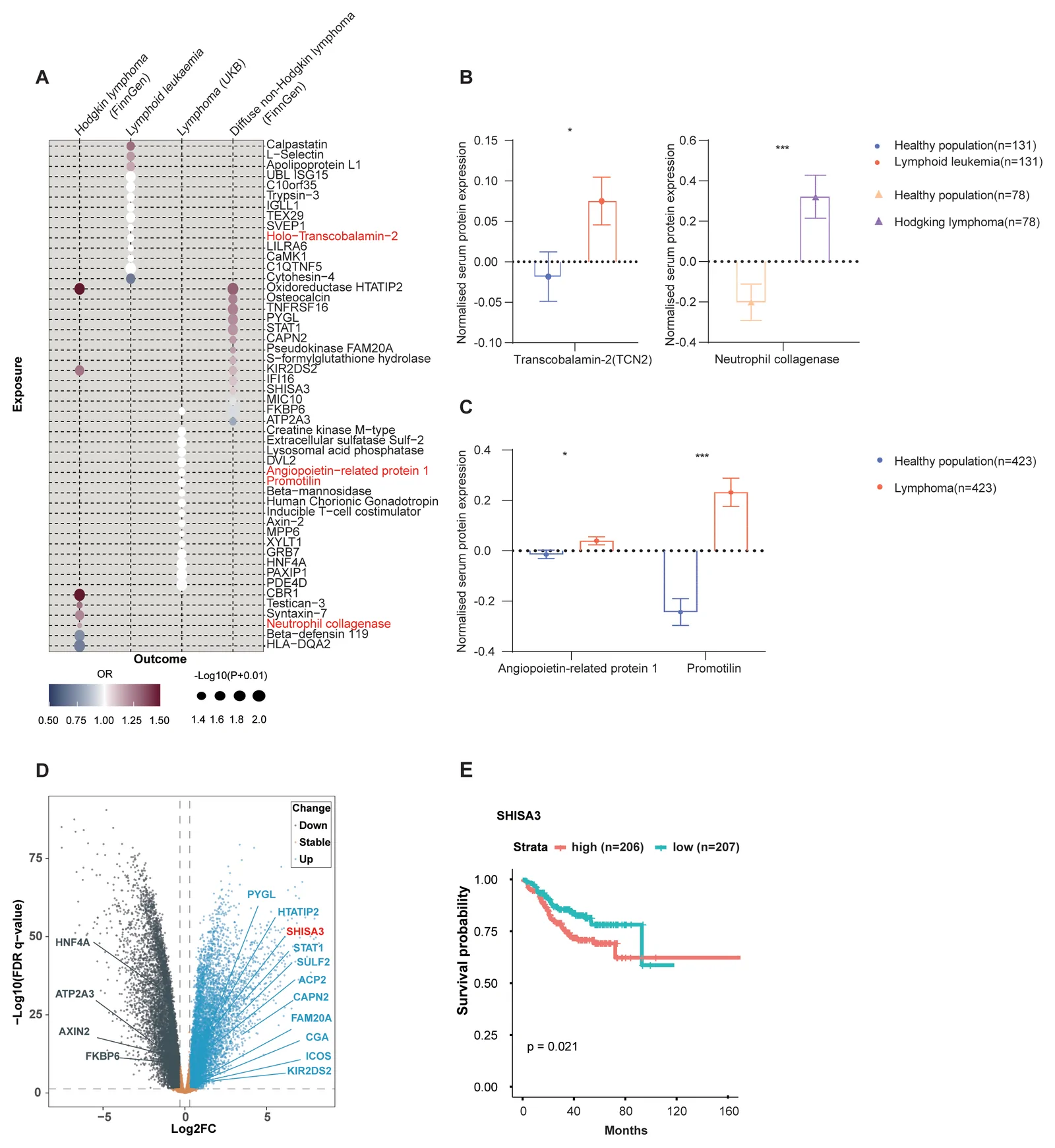
Identification of plasma proteins causally associated with LM. **(A)** Causal relationships between plasma proteins and LM are elucidated, with the complete dataset available in **Supplementary Table 1**. Circular size signifies -Log10(P + 0.01) values, color-coding corresponds to OR values. Red indicates an OR value > 1, with darker shades denoting larger OR values; blue signifies an OR value < 1, with darker shades representing smaller OR values. OR larger than 1.5 is set as 1.5, while OR smaller than -0.5 will be set as -0.5. **(B, C)** The barplot illustrates the change in levels of the risk factor for LM. **(D)** The volcano plot demonstrating the genes of interest. The color of the label indicates the statistical differences. The gene highlighted in red is a gene associated with prognosis. **(E)** Survival curve of the risk factors for lymphoma. * Represents *p* < 0.05, ** means *p* < 0.01 while *** equals *p* < 0.001. UBL ISG15:Ubiquitin-like protein ISG15; C10orf35:Uncharacterized protein C10orf35; IGLL1:Immunoglobulin lambda-like polypeptide 1; TEX29:Testis-expressed sequence 29 protein; SVEP1:Sushi, von Willebrand factor type A, EGF and pentraxin domain-containing protein 1; LILRA6:Leukocyte immunoglobulin-like receptor subfamily A member 6; CaMK1:Calcium/calmodulin-dependent protein kinase type 1; C1QTNF5:Complement C1q tumor necrosis factor-related protein 5; TNFRSF16: Tumor necrosis factor receptor superfamily member 16; PYGL: Glycogen phosphorylase, liver form; STAT1:Signal transducer and activator of transcription 1-alpha/beta; CAPN2:Calpain-2 catalytic subunit; KIR2DS2:Killer cell immunoglobulin-like receptor 2DS2; IFI16: Gamma-interferon-inducible protein 16; SHISA3: Protein shisa-3 homolog; MIC10: MICOS complex subunit MIC10; FKBP6: Inactive peptidyl-prolyl cis-trans isomerase FKBP6;ATP2A3:Sarcoplasmic/endoplasmic reticulum calcium ATPase 3; DVL2:Segment polarity protein dishevelled homolog DVL-2; MPP6:MAGUK p55 subfamily member 6; XYLT1:Xyloside xylosyltransferase 1; GRB7:Growth factor receptor-bound protein 7; HNF4A: Hepatocyte nuclear factor 4-alpha;PAXIP1: PAX-interacting protein 1; PDE4D:cAMP-specific 3’,5’-cyclic phosphodiesterase 4D; CBR1: Carbonyl reductase [NADPH] 1; HLA−DQA2:HLA class II histocompatibility antigen, DQ alpha 2 chain.

Next, we analyzed the causal relationship between plasma proteins and LM, using 2,568 GWAS traits related to plasma proteins in the IEU database. Fifty proteins associated with LM were identified, including 14 related to lymphoid leukemia and 36 to lymphoma (**Supplementary Table 1**). Genetically elevated levels of 12 plasma proteins were associated with increased risk, while 2 plasma proteins were associated with a decreased risk of lymphoid leukemia. Twenty-four plasma proteins function causally as risk factors, while 12 function as protective factors for lymphoma. Notably, some risk plasma proteins increase susceptibility to both HL and DLBCL, such as oxidoreductase HTATIP2 and killer cell immunoglobulin-like receptor 2DS2 (**Figure 3A**).

To validate the Mendelian randomization results, we analyzed the expression of these plasma proteins between healthy individuals and patients with lymphocytic leukemia and lymphoma using the Olink datasets in the UK Biobank. Consistent with the Mendelian randomization results, patients with lymphoid leukemia exhibited elevated levels of transcobalamin-2 compared to healthy individuals (**Figure 3B**). Additionally, plasma levels of angiopoietin-related protein 1 and promotilin were significantly higher in lymphoma patients, and neutrophil collagenase was found to be upregulated in patients with HL (**Figures 3B,C**). We further validated the risk factors identified by Mendelian randomization through transcriptomics studies in clinical cohorts. In diffuse large B-cell lymphoma, we observed that the expression of most risk factors was upregulated, while the majority of protective factors were downregulated (**Figure 3D**).

Since these results confirmed the roles of these proteins in LM pathogenesis, we next analyzed whether these proteins were also associated with clinical treatment outcomes for LM. Notably, Kaplan–Meier analysis of survival cohort data from our center showed that higher *SHISA3* levels were associated with poorer survival in patients with lymphoma (log-rank *P* = 0.021; **Figure 3E**), suggesting that *SHISA3* may be relevant not only to lymphoma pathogenesis but also to disease progression.

### Dysregulated lipid and amino acid metabolism constitutes a causal metabolic signature of LM

**Figure 4:**
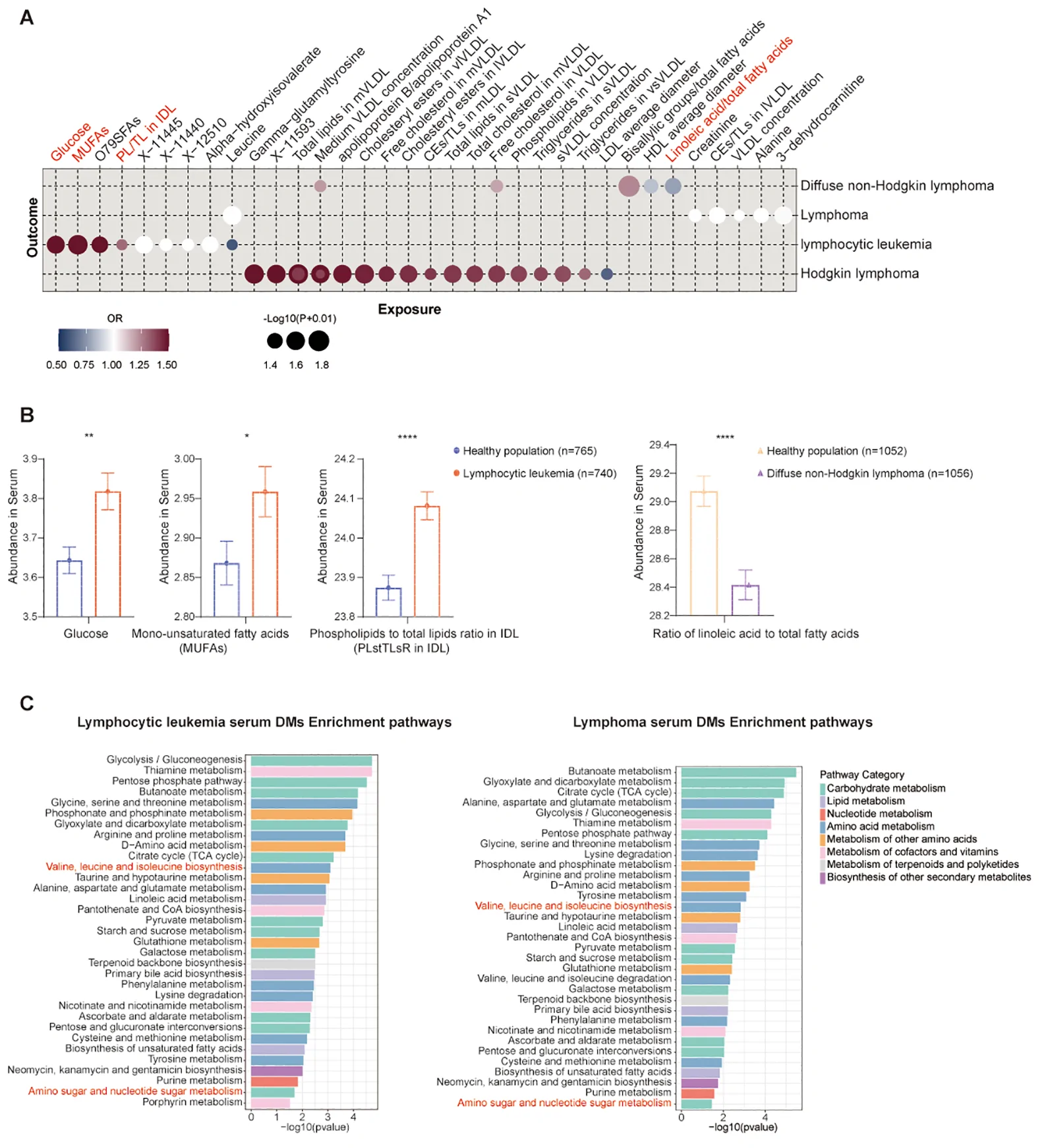
Deciphering of plasma metabolites causally related to LM. **(A)** Depicts the causal relationship identified by MR between plasma metabolites and lymphoid malignancies. The complete table is listed in **Supplementary Table 1**. The circular size represents -Log10(P + 0.01) values, with colors indicating OR **(Odds Ratio)** values. Red signifies OR > 1, with darker shades indicating larger OR values, while blue indicates OR < 1, with darker shades indicating smaller OR values. OR larger than 1.5 is set as 1.5, while OR smaller than 0.5 will be set as 0.5. **(B)** Barplot illustrating the metabolites of interest in the plasma metabonomic of LM from the UK Biobank. **(C)** Pathway analysis of differential metabolites of LM. * represents *p* < 0.05, ** means *p* < 0.01, *** equals *p* < 0.001 while **** indicates *p* < 0.0001. MUFAs: Mono-unsaturated fatty acids; O79SFAs: Omega-7, omega-9 and saturated fatty acids; PL/TL in IDL: Phospholipids to total lipids ratio in IDL; X-11445:X-11445--5-alpha-pregnan-3beta,20alpha-disulfate;X-12510:X-12510--2-aminooctanoic acid; X-11593: X-11593--O-methylascorbate*; Total lipids in mVLDL: Total lipids in medium VLDL; Cholesteryl esters in vlVLDL: Cholesteryl esters in very large VLDL; Free cholesterol in mVLDL: Free cholesterol in medium VLDL;.

Emerging evidence suggests that metabolic reprogramming significantly contributes to malignant diseases. We thus explored the causal effect of plasma metabolites on LM, using 662 GWAS traits related to plasma metabolites in the IEU database. Nine plasma metabolites were identified to exert a causal effect on lymphoid leukemia, and 26 metabolites were determined to be causally associated with the occurrence of lymphoma (**Supplementary Table 1**). Genetically elevated levels of distinct lipids in lipoproteins were observed in HL, such as total lipids, free cholesterol, or total cholesterol in medium very-low-density lipoprotein (mVLDL), and total cholesterol, phospholipids, or free cholesterol in very-low-density lipoprotein (VLDL). Additionally, in DLBCL, we found that medium VLDL concentration and the ratio of bisallylic groups to total fatty acids increased the risk of the disease, while the ratio of linoleic acid to total fatty acids was associated with a decreased risk of DLBCL (**Figure 4A**). In lymphoid leukemia, we found that glucose, monounsaturated fatty acids (MUFAs), omega-7, omega-9, and saturated fatty acids (O79SFAs), and the phospholipids to total lipids ratio in intermediate density lipoprotein (PL/TL in IDL) significantly increased the disease risk. Interestingly, we also found abnormalities in amino acid metabolism, such as leucine reducing the risk of lymphoid leukemia and alanine similarly reducing the risk of lymphoma.

Next, we used metabolomics data from the UK Biobank to validate some of the MR analysis results. We found that glucose and MUFAs were upregulated in patients with lymphoid leukemia. Compared to healthy controls, these leukemia patients exhibited an elevated phospholipids to total lipids ratio in intermediate density lipoprotein (IDL). Moreover, the protective factor against DLBCL, the ratio of linoleic acid to total fatty acids, was significantly downregulated in these patients. Given the convergence of the MR evidence identifying leucine as a protective metabolite, its biological plausibility as a metabolic regulator, and its potential translational accessibility, we subsequently prioritized leucine for functional and mechanistic validation.

We further performed pathway analysis for the differential metabolites associated with lymphoid leukemia and lymphoma, respectively. In addition to lipid metabolism pathways, amino sugar and nucleotide sugar metabolism and valine, leucine, and isoleucine biosynthesis were involved in the two malignant diseases (**Figure 4C**).

### Promotilin, glucose, and MUFAs mediate the autoimmune-driven risk of lymphoid malignancies

**Figure 5:**
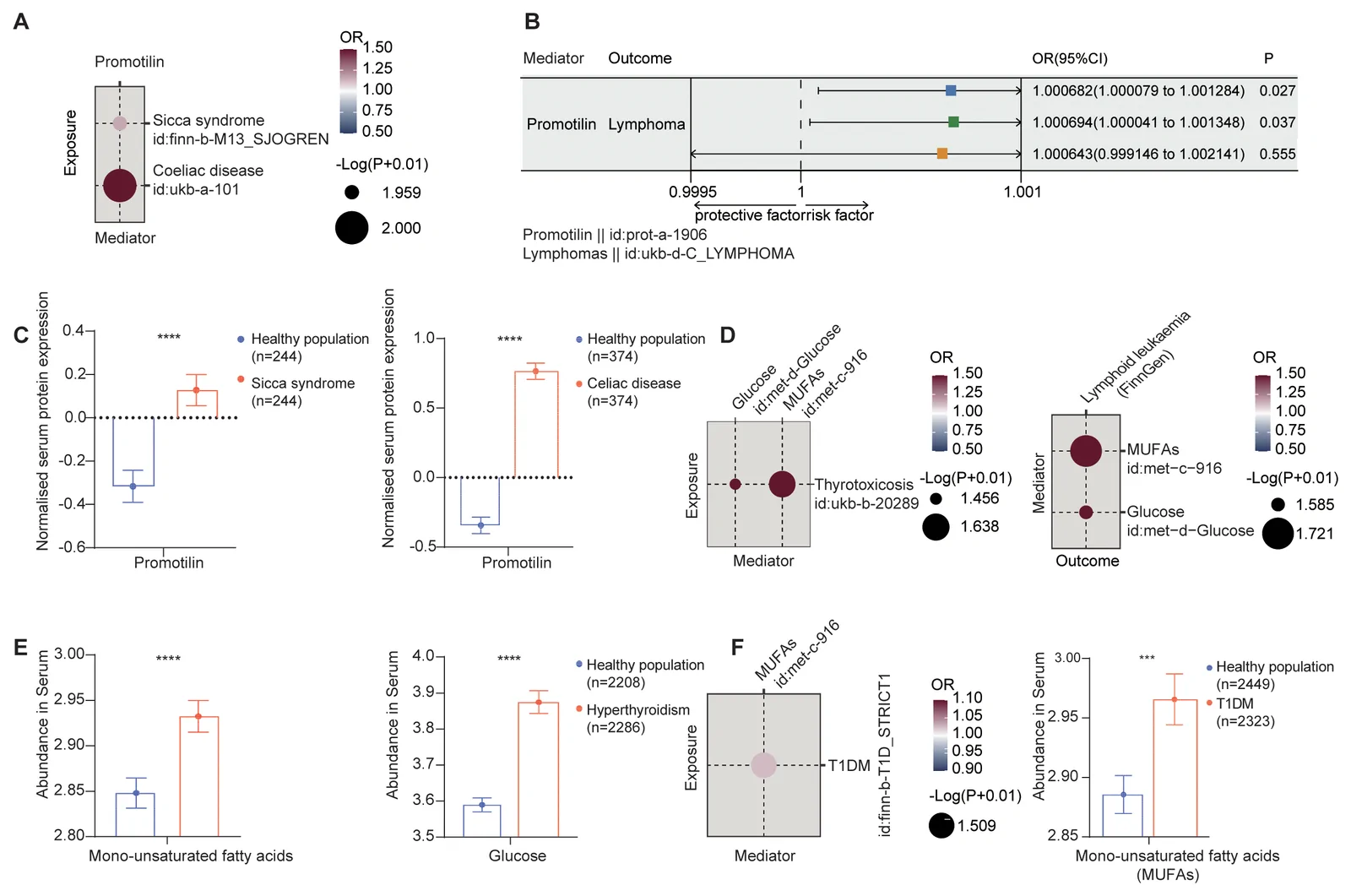
Identification of mediators for LM driven by autoimmune diseases. **(A)** Depicts the causal relationship between two autoimmune diseases and promotilin. The circular size represents -Log10(P + 0.01) values, with colors indicating OR **(Odds Ratio)** values. Red signifies OR > 1, with darker shades indicating larger OR values, while blue indicates OR < 1, with darker shades indicating smaller OR values. OR larger than 1.5 is set as 1.5, while OR smaller than 0.5 will be set as 0.5. **(B)** Forest plot delineating the positive association between circulating promotilin and lymphoma. The corresponding ID is shown in the lower left corner **(C)** Barplot illustrating the change in plasma levels of promotilin in the two autoimmune diseases. **(D)** Heatmap depicting the causal relationship between hyperthyroidism and two mediator metabolites and the latter and lymphoid leukemia. **(E)** Barplot illustrating the change in plasma levels of mediator metabolites in hyperthyroidism patients. **(F)** Depicts the causal relationship between the T1DM and mono-unsaturated fatty acids, and the change in plasma levels of the latter in T1DM. * represents *p* < 0.05, ** means *p* < 0.01, *** equals *p* < 0.001 while **** indicates *p* < 0.0001.

Next, to unravel the underlying driving mechanism of LM pathogenesis triggered by autoimmune diseases, based on plasma protein and metabolite data, we analyzed the mediators between LM and autoimmune diseases. We first systematically scrutinized the causal relationships of a total of 2,568 plasma proteins and 662 plasma metabolites with LM. Then, we appraised the causal effect of autoimmune diseases on these plasma proteins and metabolites, aiming to identify the driving mediators. We identified promotilin, a plasma protein that may mediate lymphomagenesis in patients with Sicca syndrome or coeliac disease (**Figure 1A**). Genetically predicted Sicca syndrome or coeliac disease is associated with an elevated plasma level of promotilin, and genetically elevated plasma promotilin had a causal effect on increased susceptibility to lymphoma. Through plasma proteomics, we observed an elevated plasma level of promotilin in both these autoimmune diseases and lymphoma (**Figures 5A–C**, **Figure 3D**).

Additionally, we found that two genetically elevated plasma metabolites, mono-unsaturated fatty acids and glucose, were causally associated with an increased risk of lymphoid leukemia and served as downstream causal outcomes of hyperthyroidism. Further plasma metabolomics analysis validated the elevated levels of mono-unsaturated fatty acids and glucose in patients with hyperthyroidism and lymphoid leukemia (**Figures 5D,E**, **Figure 4B**). Finally, we found that the risk factor, mono-unsaturated fatty acids, also functions as the driving mediator for lymphoid leukemia in patients with T1DM (**Figure 5F**).

### Leucine demonstrates therapeutic efficacy and prognostic value in LM through metabolic reprogramming

**Figure 6:**
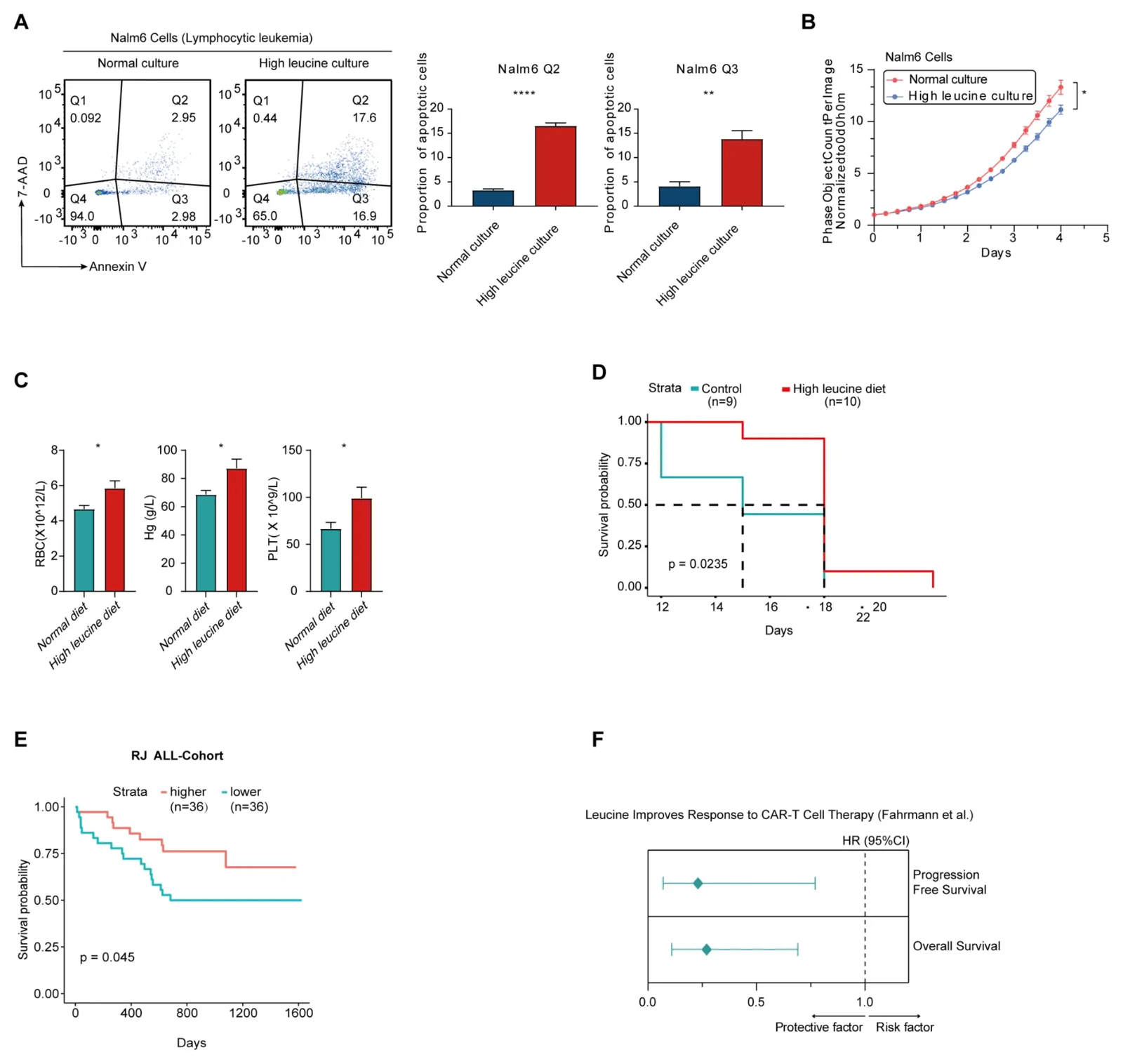
Identification of leucine as a potent protective factor for LM. **(A)** Analysis of apoptosis in NALM-6 cells under high leucine (550 mg/L) and normal culture conditions (50 mg/L). Following labeling with Annexin-V antibody and 7-AAD, cell apoptosis was assessed through flow cytometry. Q2 indicates late-stage apoptotic or dead cells while Q3 represents early-stage apoptotic cells. The results of three independent experiments are shown on the right as mean ± standard error of the mean (SEM). **(B)** Analysis of the proliferation of NALM-6 cells under the high leucine (550 mg/L) and normal culture conditions (50 mg/L). The count of cells in each image was recorded and analyzed by the 4647 Incucyte® S3 Live Cell Analysis System, Sartorius. The cell counts at each time point were normalized to the cell count at the starting time. The results of three independent experiments are displayed as mean ± SEM. **(C)** Blood routine analysis for MH/NRAS^G12D^ BCP-ALL mice. The statistical results were shown by mean with SEM. Statistical significance was determined using a t-test. **(D)** Survival analysis of MH/NRAS^G12D^ BCP-ALL mice fed with high leucine (5× leucine) (n=10) diet or normal diet (n=9). The vertical dashed line represents the median survival time. **(E)** Survival analysis based on plasma metabolomic profiling in the B-cell acute lymphoblastic leukemia **(B-ALL)** cohort from Ruijin Hospital (82). **(F)** Investigation of the relationship between leucine levels and the treatment prognosis of CAR-T-treated patients. A forest plot illustrates the hazard ratio **(HR)** and 95% confidence interval. Data sourced from previous study (88). * represents *p* < 0.05, ** means *p* < 0.01, *** equals *p* < 0.001 while **** indicates *p* < 0.0001.

To experimentally validate the protective association of leucine identified by MR and UKB analyses, we investigated its biological effects *in vitro, in vivo*, and in patient cohorts. Leucine supplementation significantly suppressed proliferation and induced apoptosis in the NALM-6 B-cell precursor acute lymphoblastic leukemia (BCP-ALL) cell line (**Figures 6A,B**). Consistent anti-tumor effects were also observed in the SU-DHL-8 lymphoma cell line, where high-leucine culture inhibited cell proliferation and promoted apoptosis (**Supplementary Figure 2****).**

To further evaluate the translational potential of leucine, we compared the effects of a high-leucine diet with a regular diet in a murine lymphocytic leukemia model. A high-leucine diet significantly prolonged survival in transplanted MEF2D-HNRNPUL1 fusion gene knock-in mice carrying the NRASG12D mutation (MH/NRASG12D BCP-ALL mice). Moreover, dietary leucine markedly alleviated anemia and thrombocytopenia in leukemic mice (**Figures 6C,D**). Consistent with these experimental findings, analysis of the Ruijin cohort demonstrated that B-ALL (non-Ph) patients with higher plasma leucine levels had significantly better prognosis than those with lower leucine levels (**Figure 6E**). Furthermore, we reanalyzed metabolomic data from a B-cell lymphoma cohort receiving CAR-T therapy and found that higher leucine levels were significantly associated with prolonged overall survival and progression-free survival in CAR-T-treated B-cell lymphoma patients (**Figure 6F****).**

**Figure 7:**
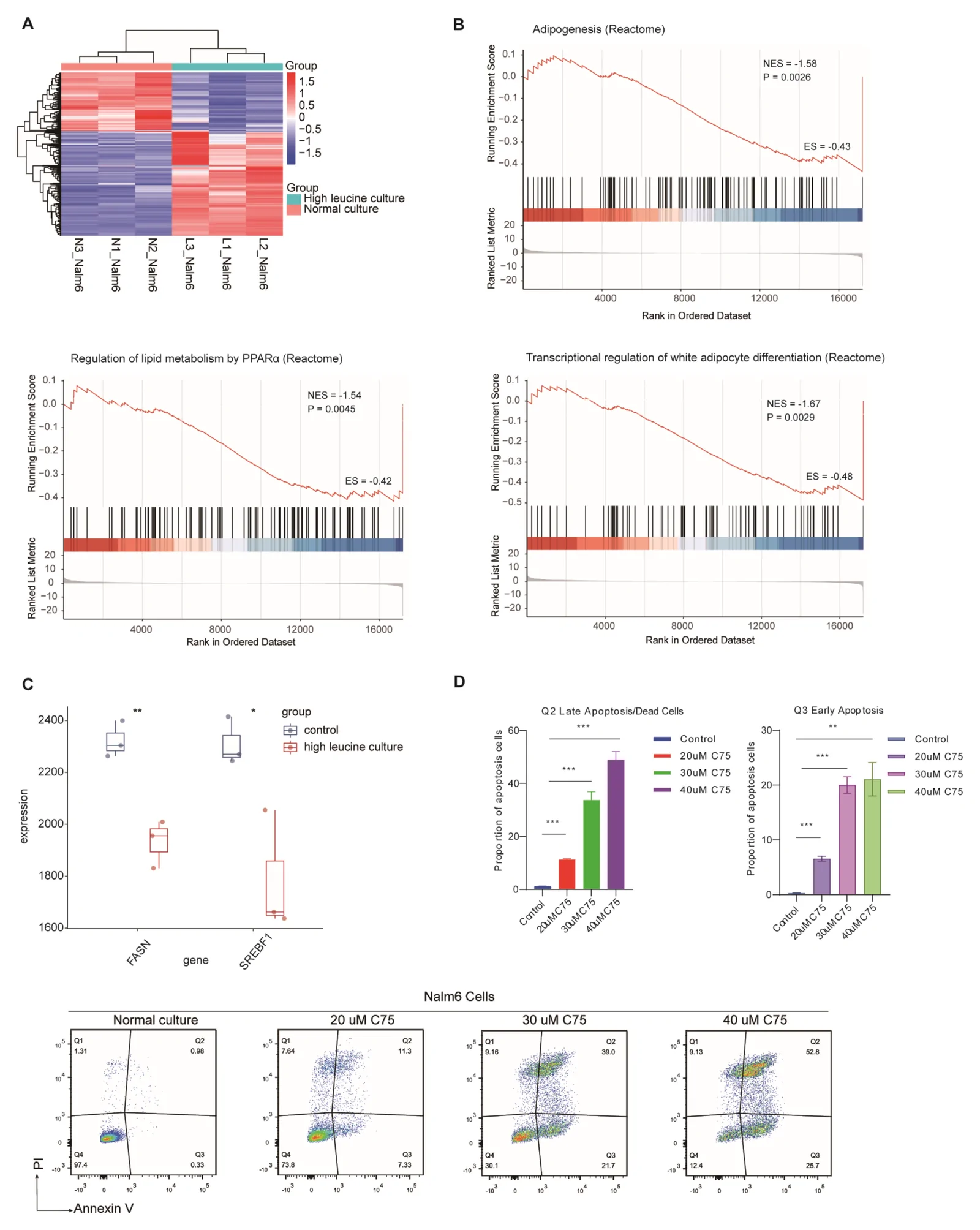
Leucine mediates anti-tumor effects by reprogramming lipid metabolism via suppressing *FASN/SREBF1* pathway. **(A)** The heatmap of differentially expressed genes (DEGs) of NALM-6 cells under normal (50 mg/L) and high leucine culture conditions (550 mg/L). **(B)** GSEA revealed suppression of adipogenesis-related pathways under high-leucine conditions (Reactome). The x-axis shows gene rank; the left and right y-axes indicate the running enrichment score and ranked list metric, respectively. **(C)** High leucine culture downregulated the mRNA expression of key lipid metabolism enzymes *SREBF1* and *FASN* in Nalm6 cells (**p < 0.01, *p < 0.05). **(D)** Annexin V flow cytometry revealed dose-dependent apoptosis in Nalm6 cells following C75 treatment (20–40 μM), as indicated by increased early (Q3) and late (Q2) apoptotic populations.

To elucidate the molecular mechanisms underlying the anti-tumor effects of leucine, we performed transcriptome sequencing of NALM-6 cells cultured under high-leucine conditions. Gene set enrichment analysis (GSEA) identified lipid metabolism as one of the most prominently suppressed biological programs, including adipogenesis (NES = -1.58, *P* = 0.0026), regulation of lipid metabolism by PPARα (NES = -1.54, *P* = 0.0045), and transcriptional regulation of white adipocyte differentiation (NES = -1.67, *P* = 0.0029) (**Figures 7A,B**). Consistently, expression of the key lipogenic regulators *SREBF1* and *FASN*, which drive *de novo* lipid synthesis, was markedly reduced under high-leucine conditions (**Figure 7C**). Functionally, pharmacological inhibition of *FASN* using C75 induced apoptosis in NALM-6 cells in a dose-dependent manner (**Figure 7D**), supporting a functional association between suppression of *de novo* lipogenesis and the anti-tumor phenotype observed following leucine exposure. Collectively, these findings suggest that *SREBF1/FASN*-mediated *de novo* lipogenesis is a potential contributory pathway, but do not establish that *SREBF1* or *FASN* is required for leucine-mediated effects.

Beyond suppression of lipid metabolism, transcriptomic profiling further revealed widespread alterations in additional metabolic and signaling pathways. Pathways involved in amino acid biosynthesis, PI3K-Akt signaling, and AGE-RAGE signaling were also significantly altered, suggesting that leucine broadly remodels cellular metabolism and intracellular signaling networks (**Supplementary Figures 3A,B**). To further explore clinically relevant downstream mediators, we evaluated leucine-regulated genes in independent leukemia cohorts from our center and the TARGET database. Eight genes upregulated by high-leucine treatment (*PLOD2, PCED1B, CLEC12A, KLHL14, NEB, NIPSNAP3B, ALOX5AP, and SPNS3*) were consistently associated with favorable prognosis in patients with lymphoid leukemia (**Supplementary Figures 3C,D**). Together, these findings indicate that leucine-induced transcriptional remodeling is not only mechanistically linked to metabolic reprogramming but also associated with improved clinical outcomes in lymphoid malignancies.

## Discussion

Elucidating the environmental and metabolic determinants of lymphoid malignancies (LM) is critical for developing novel preventive and therapeutic strategies. In this study, utilizing 10,090 GWAS datasets, large epidemiological cohorts, multiple omic datasets and experiments *in vivo* & *in vitro*, we systematically explored the risk factors for LM, particularly the causal effect of a variety of autoimmune diseases, plasma proteins and plasma metabolites on LM. Moreover, we identified that leucine is a protective factor against LM and also confers a considerably favorable prognosis for B-ALL, which may support a leucine-rich diet in high -risk population and specific leukemia patients. To our knowledge, this is the first study to systematically delineate this landscape, identifying Leucine not only as a protective dietary factor but also as a prognostic biomarker that significantly enhances the efficacy of CAR-T therapy in B-cell lymphoma patients.

Autoimmune diseases represent a group of disorders resulting from excessive activation of immunity that leads to the loss of tolerance to self-antigens. Approximately 5%–10% of the global population in the world suffer from these disorders (67), constituting a serious healthcare problem. From the perspective of genetic variation, we found that autoimmune diseases act as risk factors for LM. By contrast, LM are clonal diseases of malignant proliferation of lymphocytes, which, to some extent, are characterized by the loss or impairment of normal immune function. Of interest, although autoimmune diseases result from excessive activation of immunity, distinct types exert differential effects on LM. Some, such as UC, specifically enhance the risk of lymphoma, while others, like hyperthyroidism, increase susceptibility to both lymphoid leukemia and HL. MS, mainly prevalent in North America and Europe, was identified to have a specific causal risk on HL. We identified certain types of autoimmune diseases as novel predisposing factors in leukemogenesis, such as hyperthyroidism and membranous nephropathy.

Lipid metabolism reprogramming, often characterized by accelerated fatty acid biosynthesis, is a critical hallmark of numerous malignant tumors (68). This reprogramming is partly due to the overexpression of enzymes responsible for the synthesis of saturated fatty acids (acetyl-CoA carboxylase 1 and fatty acid synthase) and MUFAs (stearoyl-CoA desaturase, SCD1). Inhibitors of these enzymes have been validated as a potent anticancer factor *in vitro (*69–74). Abnormally elevated lipogenesis has also been observed in various hematologic malignancies (70, 75). Furthermore, we found that genetically elevated levels of multiple lipids are causally associated with an increased risk of LM, underscoring their crucial role in the pathogenesis and progression of these malignancies. Of note, we identified MUFAs as a key mediator for lymphoid leukemogenesis in patients with hyperthyroidism or T1DM. Hyperthyroidism or T1DM patients exhibit an elevated plasma level of MUFAs compared to their healthy counterparts while elevated circulating MUFAs augment susceptibility to lymphoid leukemia. Notably, MUFAs have been reported as a potent factor that protects cells against saturated fatty acid toxicity and cellular stress, thereby enhancing cell survival capability (76, 77). Moreover, the combination of bezafibrate and medroxyprogesterone acetate was validated to exert a powerful anticancer effect on patients with acute myelogenous leukemia and endemic Burkitt lymphoma through decreasing SCD1 levels and monounsaturated fatty acid synthesis (78). Collectively, we reason that the long-term dysregulated elevation of MUFAs may confer an enhanced survival ability to those abnormal lymphoid cells carrying mutated oncogenes, thereby driving the development of lymphoid leukemia in hyperthyroidism or T1DM patients.

In addition to identifying metabolic mediators underlying LM development, our study revealed plasma leucine as a protective factor against LM. Leucine is an essential branched-chain amino acid that has been implicated in regulating cellular metabolism (79), autophagy (80), and immune responses (81). Previous studies have suggested that leucine can activate antigen-presenting programs in neutrophils and enhance responses to immune checkpoint blockade in solid tumors (81), highlighting its potential role in cancer immunometabolism. However, the direct anti-leukemic effects and underlying mechanisms of leucine remain largely unclear.

Here, integrating transcriptomic profiling and functional validation, we identified metabolic reprogramming as a prominent feature associated with the anti-leukemic activity of leucine. Among the altered pathways, suppression of *de novo* lipogenesis emerged as a prominent metabolic alteration. High-leucine exposure reduced the expression of the key lipogenic regulators *SREBF1* and *FASN*, accompanied by inhibition of lipid metabolism-related transcriptional programs. Pharmacological inhibition of *FASN* using C75 phenocopied the pro-apoptotic effects of leucine, supporting a functional association between reduced lipogenesis and the anti-tumor phenotype. However, pharmacological phenocopy alone does not establish that *SREBF1* or *FASN* is required for leucine-mediated effects; genetic loss-of-function and rescue studies will be required to determine pathway necessity in the future.

Beyond lipid metabolism, leucine also induced broader transcriptional remodeling involving amino acid biosynthesis, PI3K-Akt signaling, and AGE-RAGE signaling pathways. Moreover, leucine-regulated genes identified from transcriptomic analyses were associated with favorable outcomes in independent leukemia cohorts, further supporting the clinical relevance of leucine-induced metabolic and transcriptional reprogramming. Together, these findings suggest that leucine suppresses lymphoid malignancies through coordinated metabolic remodeling, with suppression of *SREBF1/FASN*-mediated *de novo* lipogenesis representing a potential contributory mechanism rather than a definitively established mechanistic axis. Beyond its impact on cancer cells, leucine’s role extends to clinical implications. Although the metabolomics dataset from the Ruijin Hospital cohort has been previously published (82), the potential prognostic relevance of plasma leucine levels in non-Ph B-ALL patients was not explored. Our secondary analysis filled this gap, revealing that higher leucine levels are significantly associated with improved clinical outcomes, suggesting a novel prognostic biomarker. Most notably, we found that higher plasma leucine levels were associated with improved outcomes following CAR-T therapy in B-cell lymphoma patients; B-cell lymphoma patients with elevated plasma leucine levels achieved substantial prolongation in both overall and progression-free survival. These clinical associations provide a rationale for considering metabolic state alongside established determinants of T-cell function in cancer immunotherapy (83). In parallel, CAR-T and related adoptive cellular therapies have become central therapeutic strategies in hematologic malignancies, including multiple myeloma (84). Additionally, given that essential amino acids can prevent muscle loss in late-stage cancer (85), the multifaceted protective effects of leucine support the concept of nutritional adjuvant therapy. Incorporating a leucine-rich diet may offer therapeutic benefits and serve as a potential risk-reduction strategy for high-risk populations, a translational direction that warrants further exploration.

An additional unresolved question concerns the relationship between leucine sensing, mTORC1 signaling, and the *SREBF1/FASN* lipogenic program. Leucine is a well-established nutrient signal for mTORC1, with Sestrin2 functioning as an intracellular leucine sensor upstream of GATOR2/Rag GTPase-mTORC1 signaling (86, 87). The suppression of *SREBF1/FASN* observed after sustained high-leucine exposure in our model therefore cannot be assumed to reflect a simple linear leucine-mTORC1-*SREBF1/FASN* pathway. Because temporal p-S6K/p-S6 measurements and direct mTORC1 perturbation were not performed, whether the observed lipogenic suppression is mTORC1-dependent, mTORC1-independent, or an adaptive response to prolonged leucine exposure remains unresolved.

Several limitations of the mechanistic analyses should be acknowledged. First, *FASN* and *SREBF1* genetic knockdown/knockout or rescue experiments were not performed, so their absolute requirement for leucine-mediated effects has not been established. Second, the current *in vitro* design compared baseline (50 mg/L) and high-leucine (550 mg/L) conditions rather than a graded concentration series; therefore, dose dependence of *SREBF1/FASN* regulation cannot be inferred. Third, the study did not include time-course measurements of p-S6K, p-S6, and FASN or direct pharmacological/genetic interrogation of mTORC1. Future studies integrating graded leucine exposure, genetic perturbation of *SREBF1/FASN*, and temporal mTORC1 analyses will be needed to define the signaling hierarchy connecting leucine availability to lipogenic reprogramming.

## Conclusion

In conclusion, our study provides a comprehensive roadmap of the environmental and metabolic origins of LM. Our findings advocate for a paradigm shift in clinical management: prioritizing LM surveillance in patients with high-risk autoimmune disorders (particularly hyperthyroidism) and exploring Leucine supplementation as a cost-effective, accessible adjuvant therapy. Our findings support closer LM surveillance in patients with high-risk autoimmune disorders, particularly hyperthyroidism, and highlight leucine supplementation as a potentially cost-effective and accessible metabolic intervention with both preventive and therapeutic potential. In experimental models, leucine supplementation significantly prolonged survival and alleviated disease-associated anemia and thrombocytopenia, while higher circulating leucine levels were associated with more favorable outcomes in patients with B-ALL and B-cell lymphoma. However, these findings do not establish the clinical efficacy of leucine supplementation. Prospective clinical trials are therefore warranted to determine whether leucine supplementation can safely reduce LM risk in high-risk populations or improve outcomes as an adjunct to standard therapy. We acknowledge that our Mendelian randomization analysis was constrained by the availability of summary-level GWAS data, which limited the granularity of subtype-specific investigations. Future studies leveraging larger, subtype-resolved cohorts are needed to refine these causal associations. Ultimately, by integrating genetic screening with metabolic intervention, this work lays the foundation for precision metabolic oncology, where dietary and therapeutic strategies are tailored to the patient’s specific immuno-metabolic profile.

## Data Availability

The epidemiological cohorts, plasma proteomics and plasma metabolomics are available from the UK Biobank upon application (https://www.ukbiobank.ac.uk). The data for RNAseq of NALM6 cell lines generated in this study are deposited at GEO (NCBI) under accession number GSE322478. Further data supporting the findings of this study can be obtained from the corresponding author upon request.

## References

[1] Alaggio R Amador C Anagnostopoulos I Attygalle AD Araujo IBO Berti E . The 5th edition of the World Health Organization classification of haematolymphoid tumours: Lymphoid neoplasms. Leukemia. (2022) 36:1720–48. doi: 10.1038/s41375-022-01620-2 35732829 PMC9214472

[2] Siegel RL Miller KD Wagle NS Jemal A . Cancer statistics, 2023. CA Cancer J Clin. (2023) 73:17–48. doi: 10.3322/caac.21763 36633525

[3] Chhikara BS Parang K . Global cancer statistics 2022: The trends projection analysis. Chem Biol Lett. (2023) 10:451.

[4] Makishima H Bowman TV Godley LA . DDX41-associated susceptibility to myeloid neoplasms. Blood. (2023) 141:1544–52. doi: 10.1182/blood.2022017715 36455200 PMC10356560

[5] Hakkarainen M Kaaja I Douglas SPM Vulliamy T Dokal I Soulier J . The clinical picture of ERCC6L2 disease: From bone marrow failure to acute leukemia. Blood. (2023) 141:2853–66. doi: 10.1182/blood.2022019425 36952636

[6] Whitlock JA . Down syndrome and acute lymphoblastic leukaemia. Br J Haematol. (2006) 135:595–602. doi: 10.1111/j.1365-2141.2006.06337.x 17054672

[7] Dutzmann CM Spix C Popp I Kaiser M Erdmann F Erlacher M . Cancer in children with Fanconi anemia and ataxia-telangiectasia-a nationwide register-based cohort study in Germany. J Clin Oncol Off J Am Soc Clin Oncol. (2022) 40:32–9. doi: 10.1200/jco.21.01495 34597127 PMC8683217

[8] Khatri SS Kim AS . Li-Fraumeni syndrome presenting as precursor B lymphoblastic leukemia mimicking intravascular large B-cell lymphoma. Blood. (2019) 134:993. doi: 10.1182/blood.2019001407 31537539

[9] Rana I Dahlberg S Steinmaus C Zhang L . Benzene exposure and non-Hodgkin lymphoma: A systematic review and meta-analysis of human studies. Lancet Planet Heath. (2021) 5:e633–43. doi: 10.1016/s2542-5196(21)00149-2 34450064 PMC9109598

[10] Morton LM Dores GM Schonfeld SJ Linet MS Sigel BS Lam CJK . Association of chemotherapy for solid tumors with development of therapy-related myelodysplastic syndrome or acute myeloid leukemia in the modern era. JAMA Oncol. (2019) 5:318–25. doi: 10.1001/jamaoncol.2018.5625 30570657 PMC6439835

[11] Fallah M Liu X Ji J Försti A Sundquist K Hemminki K . Autoimmune diseases associated with non-Hodgkin lymphoma: A nationwide cohort study. Ann Oncol Off J Eur Soc For Med Oncol. (2014) 25:2025–30. doi: 10.1093/annonc/mdu365 25081899

[12] Skrivankova VW Richmond RC Woolf BAR Davies NM Swanson SA VanderWeele TJ . Strengthening the reporting of observational studies in epidemiology using mendelian randomisation (STROBE-MR): Explanation and elaboration. BMJ (Clinical Res Ed). (2021) 375:n2233. doi: 10.1136/bmj.n2233 34702754 PMC8546498

[13] Smith GD Ebrahim S . Mendelian randomisation at 20 years: How can it avoid hubris, while achieving more? Lancet Diabetes Endocrinol. (2024) 12:14–7. doi: 10.1016/s2213-8587(23)00348-0 38048796

[14] Emdin CA Khera AV Kathiresan S . Mendelian randomization. Jama. (2017) 318:1925–6. doi: 10.1001/jama.2017.17219 29164242

[15] Lu C Chen Q Tao H Xu L Li J Wang C . The causal effect of inflammatory bowel disease on diffuse large B-cell lymphoma: Two-sample mendelian randomization study. Front Immunol. (2023) 14:1171446. doi: 10.3389/fimmu.2023.1171446 37593734 PMC10427854

[16] Martín-Masot R Herrador-López M Navas-López VM Carmona FD Nestares T Bossini-Castillo L . Celiac disease is a risk factor for mature T and NK cell lymphoma: A mendelian randomization study. Int J Mol Sci. (2023) 24:7216. doi: 10.3390/ijms24087216 37108375 PMC10139431

[17] Jia Y Yao P Li J Wei X Liu X Wu H . Causal associations of Sjögren's syndrome with cancers: A two-sample mendelian randomization study. Arthritis Res Ther. (2023) 25:171. doi: 10.1186/s13075-023-03157-w 37715206 PMC10503000

[18] Yu J Fu L Zhang Z Ding L Hong L Gao F . Causal relationships between circulating inflammatory cytokines and diffuse large B cell lymphoma: A bidirectional mendelian randomization study. Clin Exp Med. (2023) 23:4585–95. doi: 10.1007/s10238-023-01221-y 37910257

[19] Hindes MT McElligott AM Best OG Ward MP Selemidis S Miles MA . Metabolic reprogramming, Malignant transformation and metastasis: Lessons from chronic lymphocytic leukaemia and prostate cancer. Cancer Lett. (2025) 611:217441. doi: 10.1016/j.canlet.2025.217441 39755364

[20] Ren S Zhou X Wang Z Yuan K . Amino acids metabolism: A potential target for cancer treatment. Mol Cancer. (2025) 24:307. doi: 10.1186/s12943-025-02523-3 41402797 PMC12709810

[21] Kurki MI Karjalainen J Palta P Sipilä TP Kristiansson K Donner KM . FinnGen provides genetic insights from a well-phenotyped isolated population. Nature. (2023) 613:508–18. doi: 10.1038/s41586-022-05473-8 36653562 PMC9849126

[22] Burgess S Davey Smith G Davies NM Dudbridge F Gill D Glymour MM . Guidelines for performing mendelian randomization investigations: Update for summer 2023. Wellcome Open Res. (2019) 4:186. doi: 10.12688/wellcomeopenres.15555.3 32760811 PMC7384151

[23] Bowden J Davey Smith G Burgess S . Mendelian randomization with invalid instruments: Effect estimation and bias detection through Egger regression. Int J Epidemiol. (2015) 44:512–25. doi: 10.1093/ije/dyv080 26050253 PMC4469799

[24] Bowden J Davey Smith G Haycock PC Burgess S . Consistent estimation in mendelian randomization with some invalid instruments using a weighted median estimator. Genet Epidemiol. (2016) 40:304–14. doi: 10.1002/gepi.21965 27061298 PMC4849733

[25] Stroup DF Berlin JA Morton SC Olkin I Williamson GD Rennie D . Meta-analysis of observational studies in epidemiology: A proposal for reporting. Meta-analysis of observational studies in epidemiology (MOOSE) group. Jama. (2000) 283:2008–12. doi: 10.1001/jama.283.15.2008 10789670

[26] Jess T Horvath-Puho E Fallingborg J Rasmussen HH Jacobsen BA . Cancer risk in inflammatory bowel disease according to patient phenotype and treatment: A Danish population-based cohort study. Am J Gastroenterol. (2013) 108:1869–76. doi: 10.1038/ajg.2013.249 23978954

[27] Fallah M Liu X Ji J Forsti A Sundquist K Hemminki K . Autoimmune diseases associated with non-Hodgkin lymphoma: A nationwide cohort study. Ann Oncol. (2014) 25:2025–30. doi: 10.1093/annonc/mdu365 25081899

[28] Park SK Ye BD Lee C Im JP Kim YH Kim SO . Risk and clinical characteristics of lymphoma in Korean patients with inflammatory bowel diseases: A multicenter study. J Clin Gastroenterol. (2015) 49:e11–16. doi: 10.1097/mcg.0000000000000129 24705089

[29] Jung YS Han M Park S Kim WH Cheon JH . Cancer risk in the early stages of inflammatory bowel disease in Korean patients: A nationwide population-based study. J Crohns Colitis. (2017) 11:954–62. doi: 10.1093/ecco-jcc/jjx040 28333358

[30] Olen O Smedby KE Erichsen R Pedersen L Halfvarson J Hallqvist-Everhov A . Increasing risk of lymphoma over time in Crohn's disease but not in ulcerative colitis: A Scandinavian cohort study. Clin Gastroenterol Hepatol. (2023) 21:3132–42. doi: 10.1016/j.cgh.2023.04.001 37061104

[31] Sonnenberg A Duong HT McCarty DJ El-Serag HB . Concurrence of inflammatory bowel disease with multiple sclerosis or Hodgkin lymphoma. Eur J Gastroenterol Hepatol. (2023) 35:1349–53. doi: 10.1097/meg.0000000000002657 37942756

[32] Yu J Refsum E Wieszczy P Helsingen LM Perrin V Hogden A . Risk of Malignant lymphomas in patients with inflammatory bowel disease: A population-based cohort study. BMJ Open Gastroenterol. (2023) 10:e001037. doi: 10.1136/bmjgast-2022-001037 37142293 PMC10163486

[33] Kariniemi S Rantalaiho V Virta LJ Kautiainen H Puolakka K Elfving P . Malignancies among newly diagnosed systemic lupus erythematosus patients and their survival. Lupus. (2022) 31:1750–8. doi: 10.1177/09612033221131501 36200539 PMC9709542

[34] Zhang Y Li W Zhang P Guo J Sun J Lu J . Hematological Malignancies in systemic lupus erythematosus: Clinical characteristics, risk factors, and prognosis-a case-control study. Arthritis Res Ther. (2022) 24:5. doi: 10.1186/s13075-021-02692-8 34980230 PMC8722144

[35] Bae EH Lim SY Han KD Jung JH Choi HS Kim CS . Systemic lupus erythematosus is a risk factor for cancer: A nationwide population-based study in Korea. Lupus. (2019) 28:317–23. doi: 10.1177/0961203319826672 30712493

[36] Wang LH Wang WM Lin SH Shieh CC . Bidirectional relationship between systemic lupus erythematosus and non-Hodgkin's lymphoma: A nationwide population-based study. Rheumatol (Oxford England). (2019) 58:1245–9. doi: 10.1093/rheumatology/kez011 30726952

[37] Tallbacka KR Pettersson T Pukkala E . Increased incidence of cancer in systemic lupus erythematosus: A Finnish cohort study with more than 25 years of follow-up. Scandinavian J Rheumatol. (2018) 47:461–4. doi: 10.1080/03009742.2017.1384054 29318934

[38] Kuo CF Chou IJ Rees F Grainge MJ Lanyon P Davenport G . Temporal relationships between systemic lupus erythematosus and comorbidities. Rheumatol (Oxford England). (2019) 58:840–8. doi: 10.1093/rheumatology/key335 30590795

[39] Bernatsky S Clarke AE Zahedi Niaki O Labrecque J Schanberg LE Silverman ED . Malignancy in pediatric-onset systemic lupus erythematosus. J Rheumatol. (2017) 44:1484–6. doi: 10.3899/jrheum.170179 28765255 PMC5657309

[40] Chan PC Yu CH Yeh KW Horng JT Huang JL . Comorbidities of pediatric systemic lupus erythematosus: A 6-year nationwide population-based study. J Microbiology Immunology Infection = Wei Mian Yu Gan Ran Za Zhi. (2016) 49:257–63. doi: 10.1016/j.jmii.2014.05.001 25066707

[41] Yu KH Kuo CF Huang LH Huang WK See LC . Cancer risk in patients with inflammatory systemic autoimmune rheumatic diseases: A nationwide population-based dynamic cohort study in Taiwan. Medicine. (2016) 95:e3540. doi: 10.1097/md.0000000000003540 27149461 PMC4863778

[42] Ramsey-Goldman R Brar A Richardson C Salifu MO Clarke A Bernatsky S . Standardised incidence ratios (SIRs) for cancer after renal transplant in systemic lupus erythematosus (SLE) and non-SLE recipients. Lupus Sci Med. (2016) 3:e000156. doi: 10.1136/lupus-2016-000156 27335659 PMC4908873

[43] Treppo E Toffolutti F Manfrè V Taborelli M De Marchi G De Vita S . Risk of cancer in connective tissue diseases in Northeastern Italy over 15 years. J Clin Med. (2022) 11:4272. doi: 10.3390/jcm11154272 35893361 PMC9332163

[44] Chang SH Park JK Lee YJ Yang JA Lee EY Song YW . Comparison of cancer incidence among patients with rheumatic disease: A retrospective cohort study. Arthritis Res Ther. (2014) 16:428. doi: 10.1186/s13075-014-0428-x 25163486 PMC4295295

[45] Bernatsky S Ramsey-Goldman R Labrecque J Joseph L Boivin JF Petri M . Cancer risk in systemic lupus: An updated international multi-centre cohort study. J Autoimmun. (2013) 42:130–5. doi: 10.1016/j.jaut.2012.12.009 23410586 PMC3646904

[46] Dey D Kenu E Isenberg DA . Cancer complicating systemic lupus erythematosus--a dichotomy emerging from a nested case-control study. Lupus. (2013) 22:919–27. doi: 10.1177/0961203313497118 23857987 PMC4107831

[47] Dreyer L Faurschou M Mogensen M Jacobsen S . High incidence of potentially virus-induced Malignancies in systemic lupus erythematosus: A long-term followup study in a Danish cohort. Arthritis Rheumatism. (2011) 63:3032–7. doi: 10.1002/art.30483 21953088

[48] Kang KY Kim HO Yoon HS Lee J Lee WC Ko HJ . Incidence of cancer among female patients with systemic lupus erythematosus in Korea. Clin Rheumatol. (2010) 29:381–8. doi: 10.1007/s10067-009-1332-7 20041274

[49] Chen YJ Chang YT Wang CB Wu CY . Malignancy in systemic lupus erythematosus: A nationwide cohort study in Taiwan. Am J Med. (2010) 123:1150.e1151–1156. doi: 10.1016/j.amjmed.2010.08.006 21183006

[50] DerSimonian R Laird N . Meta-analysis in clinical trials. Controlled Clin Trials. (1986) 7:177–88. doi: 10.1016/0197-2456(86)90046-2 3802833

[51] Higgins JP Thompson SG Deeks JJ Altman DG . Measuring inconsistency in meta-analyses. BMJ (Clinical Res ed). (2003) 327:557–60. doi: 10.1136/bmj.327.7414.557 12958120 PMC192859

[52] Balduzzi S Rücker G Schwarzer G . How to perform a meta-analysis with R: a practical tutorial. Evidence-Based Ment Health. (2019) 22:153–60. doi: 10.1136/ebmental-2019-300117 31563865 PMC10231495

[53] Dybkær K Bøgsted M Falgreen S Bødker JS Kjeldsen MK Schmitz A . Diffuse large B-cell lymphoma classification system that associates normal B-cell subset phenotypes with prognosis. J Clin Oncol Off J Am Soc Clin Oncol. (2015) 33:1379–88. doi: 10.1200/JCO.2014.57.7080 PMC4397280 25800755

[54] Qin W Wang N Shi Q Sun R Zheng Z Fu D . Genomic and transcriptomic characterization reveals B-cell hyperactivation and immune evasion in hepatitis B virus-associated diffuse large B-cell lymphoma. Clin Transl Med. (2023) 13:e1293. doi: 10.1002/ctm2.1293 37288581 PMC10248824

[55] Zhang MC Tian S Fu D Wang L Cheng S Yi HM . Genetic subtype-guided immunochemotherapy in diffuse large B cell lymphoma: The randomized GUIDANCE-01 trial. Cancer Cell. (2023) 41:1705–1716.e1705. doi: 10.1016/j.ccell.2023.09.004 37774697

[56] Li JF Dai YT Lilljebjörn H Shen SH Cui BW Bai L . Transcriptional landscape of B cell precursor acute lymphoblastic leukemia based on an international study of 1,223 cases. PNAS. (2018) 115:E11711–e11720. doi: 10.1073/pnas.1814397115 30487223 PMC6294900

[57] Liu YF Wang BY Zhang WN Huang JY Li BS Zhang M . Genomic profiling of adult and pediatric B-cell acute lymphoblastic leukemia. EBioMedicine. (2016) 8:173–83. doi: 10.1016/j.ebiom.2016.04.038 27428428 PMC4919728

[58] Mullighan CG Zhang J Harvey RC Collins-Underwood JR Schulman BA Phillips LA . JAK mutations in high-risk childhood acute lymphoblastic leukemia. PNAS. (2009) 106:9414–8. doi: 10.1073/pnas.0811761106 19470474 PMC2695045

[59] Therneau TM Grambsch PM . Modeling Survival Data: Extending the Cox Model. New York: Springer (2000).

[60] Therneau TM . A package for survival analysis in R. (2024).

[61] Survminer: Drawing Survival Curves Using 'Ggplot2'. Available online at: https://rpkgs.datanovia.com/survminer/index.html (Accessed September 7, 2026).

[62] Love MI Huber W Anders S . Moderated estimation of fold change and dispersion for RNA-seq data with DESeq2. Genome Biol. (2014) 15:550. doi: 10.1186/s13059-014-0550-8 25516281 PMC4302049

[63] Tang D Chen M Huang X Zhang G Zeng L Zhang G . SRplot: A free online platform for data visualization and graphing. PloS One. (2023) 18:e0294236. doi: 10.1371/journal.pone.0294236 37943830 PMC10635526

[64] Burrows K Haycock P . Genome-wide association study of cancer risk in UK biobank. (2021).

[65] Gunaseelan S Prakash A . Thalidomide-induced stroke in a child with thalassemia major. J Pediatr Hematology/Oncology. (2017) 39:e519–20. doi: 10.1097/mph.0000000000000860 28538510

[66] Warren JT Link DC . Clonal hematopoiesis and risk for hematologic Malignancy. Blood. (2020) 136:1599–605. doi: 10.1182/blood.2019000991 32736382 PMC8209630

[67] Wang L Wang FS Gershwin ME . Human autoimmune diseases: a comprehensive update. J Internal Med. (2015) 278:369–95. doi: 10.1111/joim.12395 26212387

[68] Menendez JA Lupu R . Fatty acid synthase and the lipogenic phenotype in cancer pathogenesis. Nat Rev Cancer. (2007) 7:763–77. doi: 10.1038/nrc2222 17882277

[69] Noto A Raffa S De Vitis C Roscilli G Malpicci D Coluccia P . Stearoyl-CoA desaturase-1 is a key factor for lung cancer-initiating cells. Cell Death Dis. (2013) 4:e947. doi: 10.1038/cddis.2013.444 24309934 PMC3877537

[70] Pizer ES Wood FD Pasternack GR Kuhajda FP . Fatty acid synthase (FAS): a target for cytotoxic antimetabolites in HL60 promyelocytic leukemia cells. Cancer Res. (1996) 56:745–51. 8631008

[71] Beckers A Organe S Timmermans L Scheys K Peeters A Brusselmans K . Chemical inhibition of acetyl-CoA carboxylase induces growth arrest and cytotoxicity selectively in cancer cells. Cancer Res. (2007) 67:8180–7. doi: 10.1158/0008-5472.can-07-0389 17804731

[72] Fritz V Benfodda Z Rodier G Henriquet C Iborra F Avancès C . Abrogation of de novo lipogenesis by stearoyl-CoA desaturase 1 inhibition interferes with oncogenic signaling and blocks prostate cancer progression in mice. Mol Cancer Ther. (2010) 9:1740–54. doi: 10.1201/9780429179587-21 PMC3315476 20530718

[73] Kant S Kumar A Singh SM . Fatty acid synthase inhibitor orlistat induces apoptosis in T cell lymphoma: role of cell survival regulatory molecules. Biochim Biophys Acta. (2012) 1820:1764–73. doi: 10.1016/j.bbagen.2012.07.010 22877747

[74] Wang C Xu C Sun M Luo D Liao DF Cao D . Acetyl-CoA carboxylase-alpha inhibitor TOFA induces human cancer cell apoptosis. Biochem Biophys Res Commun. (2009) 385:302–6. doi: 10.1016/j.bbrc.2009.05.045 19450551 PMC2724073

[75] Bhatt AP Jacobs SR Freemerman AJ Makowski L Rathmell JC Dittmer DP . Dysregulation of fatty acid synthesis and glycolysis in non-Hodgkin lymphoma. PNAS. (2012) 109:11818–23. doi: 10.1073/pnas.1205995109 22752304 PMC3406848

[76] Listenberger LL Han X Lewis SE Cases S Farese RV Jr. Ory DS . Triglyceride accumulation protects against fatty acid-induced lipotoxicity. PNAS. (2003) 100:3077–82. doi: 10.1073/pnas.0630588100 12629214 PMC152249

[77] Nolan CJ Larter CZ . Lipotoxicity: why do saturated fatty acids cause and monounsaturates protect against it? J Gastroenterol Hepatol. (2009) 24:703–6. doi: 10.1111/j.1440-1746.2009.05823.x 19646010

[78] Southam AD Khanim FL Hayden RE Constantinou JK Koczula KM Michell RH . Drug redeployment to kill leukemia and lymphoma cells by disrupting SCD1-mediated synthesis of monounsaturated fatty acids. Cancer Res. (2015) 75:2530–40. doi: 10.1158/0008-5472.can-15-0202 25943877

[79] Takeuchi S Nawashiro H Wada K Nomura N Toyooka T Otani N . L-Leucine induces growth arrest and persistent ERK activation in glioma cells. Amino Acids. (2012) 43:717–24. doi: 10.1007/s00726-011-1122-9 22009140

[80] Zheng Z Yan G Li X Fei Y Sun L Yu H . Lysine crotonylation regulates leucine-deprivation-induced autophagy by a 14-3-3epsilon-PPM1B axis. Cell Rep. (2022) 41:111850. doi: 10.1016/j.celrep.2022.111850 36543144

[81] Wu Y Ma J Yang X Nan F Zhang T Ji S . Neutrophil profiling illuminates anti-tumor antigen-presenting potency. Cell. (2024) 187:1422–1439.e1424. doi: 10.1016/j.cell.2024.02.005 38447573

[82] Wang JY Gui TT Jiao B Liu X Ma XL Wang C . Metabolomic insights into pathogenesis and therapeutic potential in adult acute lymphoblastic leukemia. Proc Natl Acad Sci USA. (2025) 122:e2423169122. doi: 10.1073/pnas.2423169122 39946534 PMC11848409

[83] Pu J Liu T Zhou Y Chen M Fu X Wan Y . T cells in cancer: mechanistic insights and therapeutic advances. biomark Res. (2025) 13:97. doi: 10.1186/s40364-025-00807-w 40660400 PMC12261716

[84] Pu J Liu T Sharma A Jiang L Wei F Ren X . Advances in adoptive cellular immunotherapy and therapeutic breakthroughs in multiple myeloma. Exp Hematol Oncol. (2024) 13:105. doi: 10.1186/s40164-024-00576-6 39468695 PMC11514856

[85] Engelen M Safar AM Bartter T Koeman F Deutz NEP . High anabolic potential of essential amino acid mixtures in advanced nonsmall cell lung cancer. Ann Oncol. (2015) 26:1960–6. doi: 10.1093/annonc/mdv271 26113648 PMC4551158

[86] Wolfson RL Chantranupong L Saxton RA Shen K Scaria SM Cantor JR . Sestrin2 is a leucine sensor for the mTORC1 pathway. Science. (2016) 351:43–8. doi: 10.1126/science.aab2674 26449471 PMC4698017

[87] Saxton RA Knockenhauer KE Wolfson RL Chantranupong L Pacold ME Wang T . Structural basis for leucine sensing by the Sestrin2-mTORC1 pathway. Science. (2016) 351:53–8. doi: 10.1126/science.aad2087 26586190 PMC4698039

[88] Fahrmann JF Saini NY Chia-Chi C Irajizad E Strati P Nair R . A polyamine-centric, blood-based metabolite panel predictive of poor response to CAR-T cell therapy in large B cell lymphoma. Cell Rep Med. (2022) 3:100720. doi: 10.1016/j.xcrm.2022.100720 36384092 PMC9729795

